# New insights into valve-related intramural and intracellular bacterial diversity in infective endocarditis

**DOI:** 10.1371/journal.pone.0175569

**Published:** 2017-04-14

**Authors:** Andreas Oberbach, Nadine Schlichting, Stefan Feder, Stefanie Lehmann, Yvonne Kullnick, Tilo Buschmann, Conny Blumert, Friedemann Horn, Jochen Neuhaus, Ralph Neujahr, Erik Bagaev, Christian Hagl, Maximilian Pichlmaier, Arne Christian Rodloff, Sandra Gräber, Katharina Kirsch, Marcus Sandri, Vivek Kumbhari, Armirhossein Behzadi, Amirali Behzadi, Joao Carlos Correia, Friedrich Wilhelm Mohr, Maik Friedrich

**Affiliations:** 1 Department of Diagnostics, Fraunhofer Institute for Cell Therapy and Immunology, Leipzig, Germany; 2 University of Leipzig/Heart Center Leipzig, Department of Cardiac Surgery, Leipzig, Germany; 3 Department of Medicine and Division of Gastroenterology and Hepatology. The Johns Hopkins Medical Institutions, Baltimore, Maryland, United States of America; 4 Institute of Clinical Immunology, University of Leipzig, Leipzig, Germany; 5 Department of Urology, University of Leipzig, Leipzig, Germany; 6 Carl Zeiss Microscopy GmbH, Global Sales Support Life Sciences Microscopy Labs Munich, Munich, Germany; 7 Department of Cardiac Surgery, Ludwig-Maximilians-University, Munich, Germany; 8 Institute for Medical Microbiology and Epidemiology of Infectious Diseases, Leipzig University Hospital, Leipzig, Germany; 9 University of Leipzig, Heart Centre, Department of Internal Medicine/Cardiology, Leipzig, Germany; Purdue University, UNITED STATES

## Abstract

**Aims:**

In infective endocarditis (IE), a severe inflammatory disease of the endocardium with an unchanged incidence and mortality rate over the past decades, only 1% of the cases have been described as polymicrobial infections based on microbiological approaches. The aim of this study was to identify potential biodiversity of bacterial species from infected native and prosthetic valves. Furthermore, we compared the ultrastructural micro-environments to detect the localization and distribution patterns of pathogens in IE.

**Material and methods:**

Using next-generation sequencing (NGS) of 16S rDNA, which allows analysis of the entire bacterial community within a single sample, we investigated the biodiversity of infectious bacterial species from resected native and prosthetic valves in a clinical cohort of 8 IE patients. Furthermore, we investigated the ultrastructural infected valve micro-environment by focused ion beam scanning electron microscopy (FIB-SEM).

**Results:**

Biodiversity was detected in 7 of 8 resected heart valves. This comprised 13 bacterial genera and 16 species. In addition to 11 pathogens already described as being IE related, 5 bacterial species were identified as having a novel association. In contrast, valve and blood culture-based diagnosis revealed only 4 species from 3 bacterial genera and did not show any relevant antibiotic resistance. The antibiotics chosen on this basis for treatment, however, did not cover the bacterial spectra identified by our amplicon sequencing analysis in 4 of 8 cases. In addition to intramural distribution patterns of infective bacteria, intracellular localization with evidence of bacterial immune escape mechanisms was identified.

**Conclusion:**

The high frequency of polymicrobial infections, pathogen diversity, and intracellular persistence of common IE-causing bacteria may provide clues to help explain the persistent and devastating mortality rate observed for IE. Improved bacterial diagnosis by 16S rDNA NGS that increases the ability to tailor antibiotic therapy may result in improved outcomes.

## Introduction

Infective endocarditis (IE) of native and prosthetic heart valves is a severe inflammatory disease of the endocardium, affecting valve structure and function and leading to death if left untreated [1–8]. Identifying the causative microorganism(s) is crucial for patient survival, lowering the risk of recurrent prosthetic valve infection, and tailoring the type and duration of antibiosis [9, 10]. Blood and valve analyses are culture-based methods and thereby restricted to those bacteria able to proliferate. It is still unclear whether bacteria cultured from blood represent the authentic pathogenic spectra of infected valves.

There are significant challenges in detecting the culprit microbe responsible for IE. Culture-based methods have limited sensitivity, especially when antibiotic therapy has already been initiated or when fastidious or slowly growing microorganisms are involved [11]. In fact, country-specific clinical studies revealed that blood culture failed to detect a species in 10–48% of patients [6, 12, 13].

The origin of pathogens responsible for IE is primarily thought to stem from systemic bacteremia as a result of lesions or surgical interventions of skin or mucosa, leading to barrier leakage and bacterial invasion. If so, one would expect that bacterial diversity would result in polymicrobial infections. Interestingly, however, polymicrobial infections have been described in only 1% of cases [6]. This is clinically significant as polymicrobial infections have been shown to reduce the rate of cure in other infectious diseases [14].

The goal of this study was to identify the pathogen spectra of infected valves using next-generation sequencing (NGS)-based 16S rDNA analysis. We aimed to:

1. identify the potential biodiversity of infectious bacterial species from native and prosthetic valves of IE patients in a clinical cohort of 8 patients;
2. analyze and compare infected native and prosthetic valve ultrastructural micro-environments by high-resolution focused ion beam scanning electron microscopy (FIB-SEM) to detect the localization and distribution patterns of pathogens.

## Material and methods

### Phenotype characteristics

The study was approved by the ethics committee of the University of Leipzig, and participants provide their written informed consent to participate in this study (286/13ff, November 2013). In our study we included 8 patients (6 men and 2 women; mean age 71.4 years; range 63–87 years, Table 1). All patients were diagnosed with IE according to the modified Duke criteria (15) as presented in Table 2. They underwent surgery for acute IE at the Heart Center Leipzig (Germany). The seven patients were randomly selected out of 57 patients by using the SPSS statistics software package. Explanted heart valves of all patients selected for our study were additionally analyzed by histological methods and diagnosed as an acute IE by expert pathologists.

**Table 1:** Phenotype.

	Valve patients
**n [male]**	8 [6]
**Age [Years]**	71.4 ± 9.04
**Height [cm]**	170.63 ± 8.55
**Weight [kg]**	90.6 ± 25.73
**BMI [kg/m^2^]**	30.71 ± 6.4
**BSA** [m^2^]	2.06 ± 0.3
**LVEF** [%]	54.38 ± 12.77
**EuroScore** [log]	46.37 ± 29.13
**EuroScore**	13.00 ± 4.96
Comorbidities	
**Hypertension** [n]	6
**CAD** [n]	3
**DMT2** [n]	2
**Valve regurgitation** [n]	8
Blood parameter	
**Creatine** [μmol]	146.45 ± 154.45
**Uric acid** [mmol/l]	9.68 ± 7.27
**ASAT** [μmol/l*s]	0.60 ± 0.18
**GGT** [μmol/l*s]	2.01 ± 20.55
**Lipase** [U/l]	31.86 ± 20.55
**Cholesterase** [μmol/l*s]	50.69 ± 29.61
**Leucocytes** [Gpt/l]	12.72 ±6.12
**CrP** [mg/l]	102.92 ± 82,83

Values shown are n or mean ± SD. Abbreviations: n = number; BMI—body-mass-index; BSA—body surface area; LVEF—left ventricular ejection fraction; EuroScore—European system for cardiac operative risk evaluation; CAD—coronary artery disease; DMT2—diabetes mellitus type 2; ASAT—aspartate aminotransferase; GGT—gamma-glutamyl transpeptidase; CrP—C-reactive protein.

**Table 2:** Clinical characteristics.

Patient no.	Native valve			Biological prosthesis				
	1	2	3	4	5	6	7	8
Valve type	TV	MV	AV	MV	AV	AV	AV	AV
**Duke Criteria**								
Major	Echo positive, BC positive	Echo positive, BC positive	Echo positive, BC positive	Echo positive, BC negative	Echo positive, BC positive	Echo positive, BC positive	Echo positive, BC positive	Echo positive, BC positive
Minor	fever, PM-Lead in RV, leg ischemia	fever, immune- suppressed	fever	fever, prosthesis, TIA	fever, prosthesis, cranial septic embolism	fever, prosthesis	fever, prosthesis	fever, prosthesis
**Standard microbiology analysis**								
Blood culture	*Staph*. *aureus*	*Enterococ*. *faecalis*	*Strep*. *gordonii*	*Ø*	*Staph*. *aureus*	*Staph*. *epidermidis*	*Staph*. *aureus*	*Enterococ*. *faecalis*
Valve swab	Ø	Ø	Ø	Ø	*Staph*. *aureus*	Ø	Ø	Ø
Valve ultra-sound	Ø	*Staph*. *epidermidis*	*Strep*. *gordonii*	Ø	*Staph*. *aureus*	Ø	Ø	*Enterococ*. *faecalis*
**Antibiotic therapy**								
Initial	Flucloxacillin Rifampicin	Tazobactam	Ø	Ampicillin/ Sulbactam Rifampicin	Vancomycin Cefuroxime	Tazobactam	Vancomycin Gentamycin Rifampicin	Ampicillin Imipenem
Extended	Cefazolin Rifampicin	Gentamycin Vancomycin Imipenem	Gentamycin Vancomycin Imipenem	Ampicillin/ Sulbactam Rifampicin	Vancomycin Cefuroxime	Vancomycin Gentamycin	Vancomycin Gentamycin Rifampicin	Vancomycin Gentamycin Rifampicin
**Mortality (postoperative within 30 days)**								
Survival	Yes	Yes	Yes	death	Yes	death	Yes	Yes
**Histological findings**								
Structural	*Staph*. *aureus*, intramural, correlated to nuclear structures	-	*Staph*. *aureus*, intramural, correlated to nuclear structures	-	*Staph*. *aureus*, intramural, correlated to nuclear structures	*Staph*. *aureus*, intramural, correlated to nuclear structures	*Staph*. *aureus*, intramural, correlated to nuclear structures	*Staph*. *aureus*, intramural, correlated to nuclear structures
Ultra-Structural	-	-	bacteria, intracellular and intramural, smooth surface structure	-	bacteria, intracellular and intramural	-	bacteria, rough surface structure	-

Abbreviations: TV—tricuspid valve; MV—mitral valve; AV—aortic valve; BC—blood culture; PM–peacemaker; RV—right ventricular; TIA—transient ischemic attack; Ø—negative; *Staph*.—*Staphylococcus*; *Enterococ*.–*Enterococcus*; *Strep*.–*Streptococcus*.

### Microbiological analysis

Clinical specimens from heart valve tissue were placed into sterile tubes filled with 6 ml of brain-heart-infusion broth (BHI) supplemented with 5% Fildes extract (both OXOID Ltd., Hampshire, England) and placed into an ultrasonic bath (BactoSonic^®^, BANDELIN electronic GmbH & Co. KG, Berlin, Germany). Sonication was performed for 15 min at an ultrasound frequency of 35 kHz to expose bacteria located in biofilms on the valve surface and to convert them into a planktonic state. Subsequently, the samples were incubated in a CO_2_-enriched atmosphere (5%) at a temperature of 37°C for 14 days. Turbidity, as evidence of bacterial growth, was assessed at days 4, 7, and 14 of incubation. The samples with suspected growth were inoculated onto 5% sheep blood-containing Columbia agar (Roth), Columbia agar supplemented with cooked blood and IsoVitalex (BD BBL, Le Pont de Claix, France), Endo-agar (Fluka, Sigma-Aldrich Chemie GmbH, Steinheim, Germany), Aesculine-agar (OXOID), and Sabouraud-2%-glucose agar plates (Sifin diagnostics GmbH, Berlin, Germany) as well as onto anaerobic media (supplemented Brucella agar (BD BBL) and incubated for a duration of 4 days under a capnophilic (5% CO_2_) and anaerobic atmosphere (GenBag, BioMérieux, Marcy LÈtoile, France) at 37°C. Blood agar plates were cross-streaked with Staphylococcus aureus H1 to enable growth of nutritionally fastidious bacteria showing satellite phenomena. Growth was evaluated daily. After 14 days of incubation, all samples were subcultured independently of whether they showed evidence of prior growth or not. If a subculture showed bacterial growth, identification was established by VITEK^®^ MALDI-ToF MS (BioMérieux Inc., Durham, NC, USA), and antimicrobial susceptibility testing was performed via the broth microdilution method or Epsilometer tests (Etest AB Biodisk, Solna, Sweden) according to the EUCAST guidelines. For long-term storage, a microbial cryopreservation stock with Cryobank^™^ (Mast Diagnostica GmbH, Reinfeld, Germany) was created for each strain.

An average of two sets of blood samples were collected during the operative setting from the heart-lung machine (HLM), central venous catheter, or by peripheral venous puncture. Samples of approximately 10 ml of blood thus collected were inoculated into aerobic and anaerobic blood culture bottles (BacT/ALERT FA/FN, BioMérieux Inc., Durham, NC, USA) and kept at room temperature. In the laboratory, blood culture bottles were placed into an automated continuous monitoring system (BacT/ALERT 3D) and incubated at 36°C for a prolonged period of 21 days. When algorithms indicated bacterial growth in a bottle, subculture was performed on the solid media as described above. Subcultures were incubated again for 4 days under a capnophilic and anaerobic atmosphere at a temperature of 36°C. Identification and antimicrobial susceptibility testing were performed as described above. In cases where bacterial growth did not occur on solid media, blood culture bottles were further incubated at 37°C and manual subculture was repeated twice during the 21 days of incubation.

### Analysis of the metagenome

To identify the bacterial spectra of infected native and prosthetic heart valves using culture-independent, genome-based analyses, total DNA was extracted from clinical specimens. Two different regions of bacterial 16S rDNA (V1-V3 and V3-V4) were amplified and analyzed using NGS to yield a maximal identifiable number of species. To test whether the discovery rate might be improved by a bacterial DNA enrichment procedure, a fraction of isolated total DNA was pretreated using the NEB Next Microbiome DNA Enrichment kit (New England BioLabs, Frankfurt am Main, Germany) prior to PCR amplification and amplicon sequencing. To determine the discovery rates of metagenome experiments, 8 randomly selected samples were subjected to repeated analysis (S3 Fig).

### DNA extraction and 16S rDNA amplification

Heart valve tissue (25 mg) was homogenized in phosphate-buffered saline, and total DNA was extracted with the QIAamp DNA Mini Kit (Qiagen, Hilden, Germany) according to the manufacturer’s instructions. Microbial DNA was enriched by using the NEBNext@ Microbiome DNA Enrichment kit. Subsequently, two hypervariable regions of bacterial 16S rRNA gene were amplified using well-established primers [15]. The following primer pairs for polymerase chain reaction (PCR) amplification were used: for the V1-V3 hypervariable region: 27F (5`-AGAGTTTGATCCTGGCTCAG-3`) and 534R (5`-ATTACCGCGGCTGCTGG-3`); for the V3-V5 hypervariable region: 357F (5`-CCTACGGGAGGCAGCAG-3`) and 926R (5`-CCGTCAATTCMTTTRAGT-3`). Briefly, each 20 μL of PCR reaction mixture contained 20 ng of total or microbial-enriched DNA as a template, 10 μL 2x Hot Start Taq Master Mix (New England BioLabs), and 0.5 μM of each primer. PCR reactions were carried out using the following protocols: (1) for the V1-V3 hypervariable region, an initial denaturation step performed at 95°C for 30 s followed by 30 cycles of denaturation (95°C, 30 s), annealing (56°C, 40 s), and extension (68°C, 1 min), and a final elongation of 5 min at 68°C; (2) for the V3-V5 hypervariable region, the parameters were the same as above except that the annealing temperature was 59°C. PCR products were visualized by agarose gel electrophoresis, showing expected product sizes (500 and 550 bp for the V1-V3 and V3-V5 regions, respectively) in all samples. Water was used as negative control in the PCR reaction. PCR products were purified using QIAquick PCR Purification Kit (Qiagen) and quantified using a NanoDrop 1000 Spectrophotometer (VWR International GmbH, Erlangen, Germany) or a Qubit 3.0 Fluorometer (Life Technologies, Darmstadt, Germany). As key control experiments, DNA was also extracted from water to ensure absence of contaminating bacterial DNA in water, buffer, equipment, and reagents. Furthermore, DNA was also extracted from valve tissue of non-infected patients (S2 Fig).

### Library preparation and next-generation sequencing

Illumina paired-end sequencing libraries were constructed from purified PCR fragments using the NEBNext^®^ DNA Library Prep Master Mix Set and Multiplex Oligos for Illumina (New England BioLabs) following standard sample preparation. One μg of purified PCR products was end-repaired, dA-tailed, ligated to platform-specific adaptors, size-selected to an average size of 500–550 bp by Agencourt AMPure XP beads (Beckman Coulter), and amplified by 4 to 8 cycles of PCR. Finally, multiplexed paired-end sequencing (2×300 bp reads) of the 16S rDNA amplicons was performed using Illumina MiSeq technology (MiSeq Reagent Kit v3 600 cycles, Illumina).

### Sequence analysis and bioinformatics

Quality of reads was controlled using fastqc (http://www.bioinformatics.babraham.ac.uk/projects/fastqc/). Adapters and low-quality bases were trimmed using Trimmomatic [16]; very short reads were removed. Remaining reads were aligned to the NCBI database 16SMicrobial using software blast in megablast mode [17], requiring a maximum e value of 0.001. Local alignment was repeated with minimum identity filters of 95%, 97%, and 99%. Blast results were then analyzed with MEGAN5 [18] in paired-end mode, requiring a minimum bit score of 60, using the NCBI taxonomy. Taxonomic classification was summarized at the species level and further analyzed using statistics software R (http://www.R-project.org/). For comparison of different samples, species and genus results were standardized as a proportion of all aligned reads. Cut-off levels of at least 0.1%, 0.5%, 1%, 2%, and 10% of species/genus-specific reads were applied (S3 Fig).

### Quantitative PCR (qPCR)

DNA of bacterial cultures *Haloplasma contractile* (DSM 18853), *Burkholderia fungorum* (DSM 17061), and *Aeribacillus pallidus* (DSM 3670) were obtained from the Leibniz Institute DSMZ-German Collection of Microorganisms and Cell Cultures (Braunschweig, Germany).

*Staphylococcus aureus*, *Enterococcus faecalis*, and *Streptococcus gordonii* were isolated from heart valve tissue and cultured as described above. After cell lysis with 20 mg/ml lysozyme (20 mM Tris·HCl, pH 8.0; 2 mM EDTA; 1.2% Triton) the bacterial DNA was extracted with the QIAamp DNA Mini Kit (Qiagen, Hilden, Germany) according to the manufacturer’s instructions. Purified DNA from monocultures was used as positive control templates in qPCR. The concentration of specific DNA dilutions was plotted against the Cp (crossing point) value to generate a standard curve used for absolute quantification.

The primers listed in Table 3 were synthesized by Eurofins Genomics (Ebersberg, Germany). PCR amplification was performed using a Roche Light Cycler 480 (Roche Diagnostics, Mannheim, Germany) with the SYBR Green fluorescence dye (QuantiNova SYBR Green PCR Kit, Qiagen, Hilden, Germany). The reaction was performed in a total volume of 20 μl. The PCR protocol consisted of an initial denaturation at 95°C for 2 min; 40 cycles of denaturation for 5 s at 95°C, and combined annealing/extension 10 s at 60°C. The serial dilution standards, negative controls, and samples were measured simultaneously in duplicate. At the end of amplification, SYBR green fluorescence was measured continuously during the melting curve by heating at 2.2°C/s increments from 65 to 95°C with continuous fluorescence monitoring. Accordingly, a melting curve was generated at the end of the PCR amplification as a measure of the specificity of the PCR reaction.

**Table 3:** Species-specific qPCR primers.

Species	Primer	Sequence (5‘-3‘)	PCR product (bp)	Reference
*Staphylococcus aureus*	Sa442-1	AAT CTT TGT CGG TAC ACG ATA TTC TTC ACG	108	[19]
	Sa442-2	CGT AAT GAG ATT TCA GTA GAT AAT ACA ACA		
*Enterococcus faecalis*	FL1_F	ACT TAT GTG ACT AAC TTA ACC	360	[20]
	FL2_R	TAA TGG TGA ATC TTG GTT TGG		
*Streptococcus gordonii*	Sgo_F2	TGT ACC CCG TAT CGT TCC TGT G	175	[21]
	Sgo_R2	AAA GAC TGG AGT TGC AAT GTG AAT A		
*Burkholderia fungorum*	FunF	GTC ATG CGG CTC GGC GCA GGT	324	[22]
	FunR	GAG TGC GTC GGC AAT CTC GAG T		
*Haloplasma contractile*	Hc_F	CGT TGA GTG CTA AGT GTC GG	185	[23]
	Hc_R	GTC AGA GGG ATG TCA AGG CT		
*Aeribacillus pallidus*	Ap_F	TCC CCT GAC AAC CCT AGA GA	174	[23]
	Ap_R	TTT AGC CGG CAG TCA CCT TA		

### Histological staining

Cryosections of heart valve tissue were fixed with 4% paraformaldehyde and incubated overnight at 4°C with anti-*Staphylococcus aureus* antibody (1:100; anti-*Staphylococcus aureus* antibody 704, ab37644, abcam, Germany). Indirect immunofluorescence was performed using secondary antibodies conjugated with Alexa Fluor 488 (1:500; Invitrogen, Life Technologies GmbH). Cell nuclei were stained with hexidium iodide (1:500; Molecular Probes, Life Technologies GmbH, Darmstadt, Germany). The tissues were analyzed with a Zeiss LSM-5 Pascal confocal laser scanning microscope (Carl Zeiss, Jena, Germany).

### Scanning electron microscopy

The heart valve tissues were cut into pieces of 2 mm^3^ and fixed with 2.5% glutaraldehyde and 2% paraformaldehyde in 0.15 M cacodylate buffer (pH 7.4) containing 2 mM calcium chloride at 35°C for 5 min. Target tissues were removed and fixed for an additional 2–3 h on ice in the same solution. They were washed 5 x 3 min in cold cacodylate buffer containing 2 mM calcium chloride. Immediately prior to use, a solution containing 3% potassium ferrocyanide in 0.3 M cacodylate buffer with 4 mM calcium chloride was combined with an equal volume of 4% aqueous osmium tetroxide. The tissues were incubated in this solution for 1 h on ice. Tissues were then washed 5 x 3 min with doubly distilled water (ddH_2_O) at room temperature and placed in filtered thiocarbohydrazide solution for 20 min at room temperature. Another washing cycle (5 x 3 min) with ddH2O at room temperature followed. Thereafter, tissues were placed in 2% osmium tetroxide in ddH20 for 30 min at room temperature. Following this second exposure to osmium, tissues were washed 5 x 3 min at room temperature in ddH2O, placed in 1% uranyl acetate (aqueous), and left in a refrigerator (~4°C) overnight. The next day, en bloc Walton’s lead aspartate staining was performed. Thus, tissues were washed 5 x 3 min in ddH2O at room temperature and then placed in the lead aspartate solution for 30 min at 60°C. After another washing cycle (5 x 3 min at room temperature in ddH_2_O), tissues were dehydrated using ice-cold solutions of freshly prepared 20%, 50%, 70%, 90%, 100%, and 100% ethanol (anhydrous) for 5 min each, placed in hexamethyldisilazane for 10 min, and dried at room temperature. Specimens were mounted on standard scanning electron microscopy (SEM) stubs with carbon tape (PELCO^®^ Isopropanol Based Graphite Paint) and stored overnight at 30°C. The cured samples were finally sputter-coated (Leica EM ACE200) with a 5–10 nm thick platinum layer and evaluated with a scanning electron microscope (Zeiss Sigma VP).

For 3View-SEM and FIB-SEM, small pieces of heart valve tissues were fixed in 2% glutaraldehyde in 0.1 M cacodylate buffer (pH7.4). Heavy metal contrast enhancement was performed according to the protocol of Deerinck et al. [24]. Specimens were embedded in especially hard epoxy resins Durcupan^™^ ACM or Araldite^®^ 506 (Sigma Aldrich, Steinheim, Germany). Micro air bubbles were removed by vacuum during resin penetration and hardening. We used a Crossbeam^®^ Auriga 60 (Zeiss) with gallium ion beam and combined SE-/ EsB-Imaging for optimal contrast. Stacks of several hundred images were automatically acquired with maximum resolution of 2.5 nm x 12.5 nm x 5 nm (xyz). For 3D reconstruction analysis we used Amira^®^ 5.0 3D Image Analysis software package (vsg, Düsseldorf, Germany) on a Mac Pro workstation (Apple Inc., Cupertino, CA, USA).

A summary of the complete workflow is depicted in S3 Fig.

## Results

### Clinical characteristics

Among the 8 cases of definitive endocarditis (Table 1), blood cultures were positive in 7 and microbiological analysis of valve tissue revealed positive results in 4 patients (Table 2). After surgery, histopathological criteria of IE were present in all definitive cases (data not shown). Blood culture analysis revealed *Staphylococcus aureus*, *Staphylococcus epidermidis*, *Streptococcus gordonii* and *Enterococcus faecalis* in 3, 1, 1 and 2 cases, respectively (Table 2).

To improve the identification of valve-related bacterial infection, we extended our protocol by utilizing a novel, ultrasound-based pretreatment procedure of the valve to extract bacteria from the tissue. The analysis of a valve swab revealed only 12.5% sensitivity (one pathogen in patient 5, *Staphylococcus aureus*) whereas ultrasound pretreatment increased the number of identified pathogens and raised the sensitivity to 50% (Table 2). The discrepancy in the results of valve swab analysis and the ultrasound-based pretreatment procedure indicates that pathogen localization may be responsible for the low sensitivity observed by microbiological standard diagnostics. Therefore, we performed FIB-SEM-based ultrastructural analysis to visualize localization of infective bacteria.

### Structural analysis using confocal fluorescence microscopy

Histological staining for *Staphylococcus aureus* in infected native (2 patients) and biological prosthetic (4 patients) valve tissues revealed its presence in all samples analyzed. (S1 Fig, Table 2) Bacteria seem to be localized intramurally as well as in proximity to eukaryotic nuclear structures (S1 Fig, Table 2).

### Ultrastructural analysis using electron microscopy

**Fig 1:**
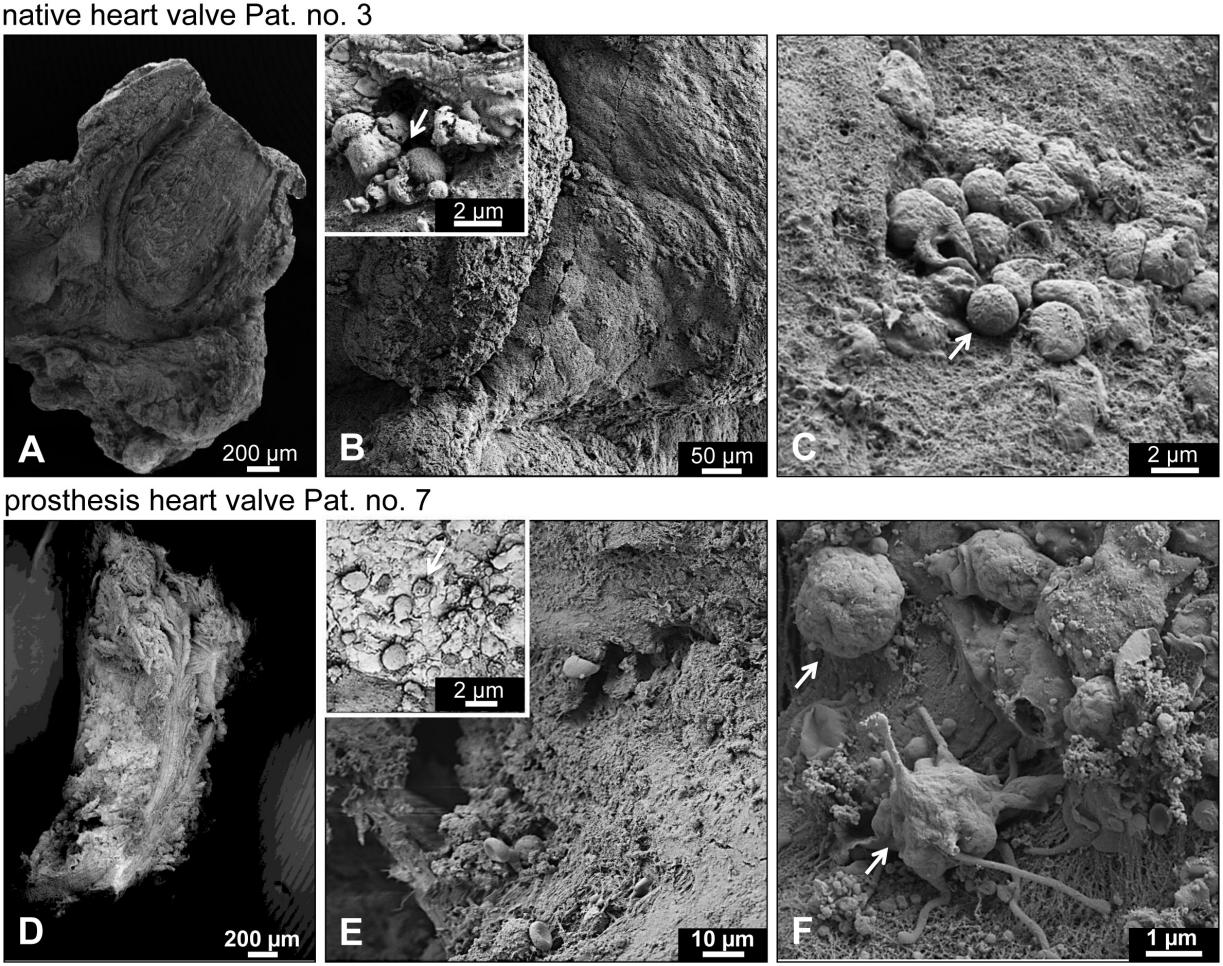
Surface analysis of infected valves. For surface analysis using scanning electron microscopy (SEM), infected native (A-C; patient 3) and prosthetic (D-F; patient 7) heart valve tissues were cut into small pieces and fixed. After pretreatment and exposure to osmium tetroxide, tissues were dehydrated and mounted on standard SEM stubs with carbon tape. The cured samples were finally sputter-coated with a platinum layer and evaluated with a scanning electron microscope. (A-C) SEM images of the surface of an infected native valve. (A) Overview. (B) Epithelial cell boundaries are discernable. Inset: note that few bacteria are attached to the smooth surface and that these seem to be often damaged (arrow). (C) At higher magnification scattered bacteria showing apparently intact morphology (arrow) can also be found. (D-F) Ultrastructure images of infected biological prosthetic valve. (D) Overview. (E) The surface is characterized by deep holes and cracks where microbes may be concealed; the surface appears rough. Inset: note that the few bacteria attached to the outside often seem to be damaged (arrow). (F) At higher magnification a number of apparently intact bacteria (arrows) showing different morphologies can be found; note the fibrous structure of the substrate, providing ideal adhesion sites.

To obtain data that may provide an explanation for the low sensitivity of microbial valve culture diagnosis, we applied sophisticated electron microscopy (FIB-SEM) to one infected native and two biological prosthetic valve specimens (Fig 1A–1D). First, ultrastructural analysis revealed a smooth surface with frequently discernable epithelial cell boundaries in the native valve (Fig 1B) and a rather rough-structured surface characterized by deep holes and cracks where microbes may be concealed (“Fig 1E and 1F”) in the prosthetic valve. Second, electron microscopy showed pathogen-like structures of 500 nm to 2 μm in diameter attached to the surface, clearly suggesting the presence of bacteria. The vast majority seem to be damaged (Fig 1B and 1E, **inset**). Using high magnification a few bacteria with potential intact morphology were also present (Fig 1C and 1F). Evidence of different bacterial morphologies was also found (Fig 1F).

**Fig 2:**
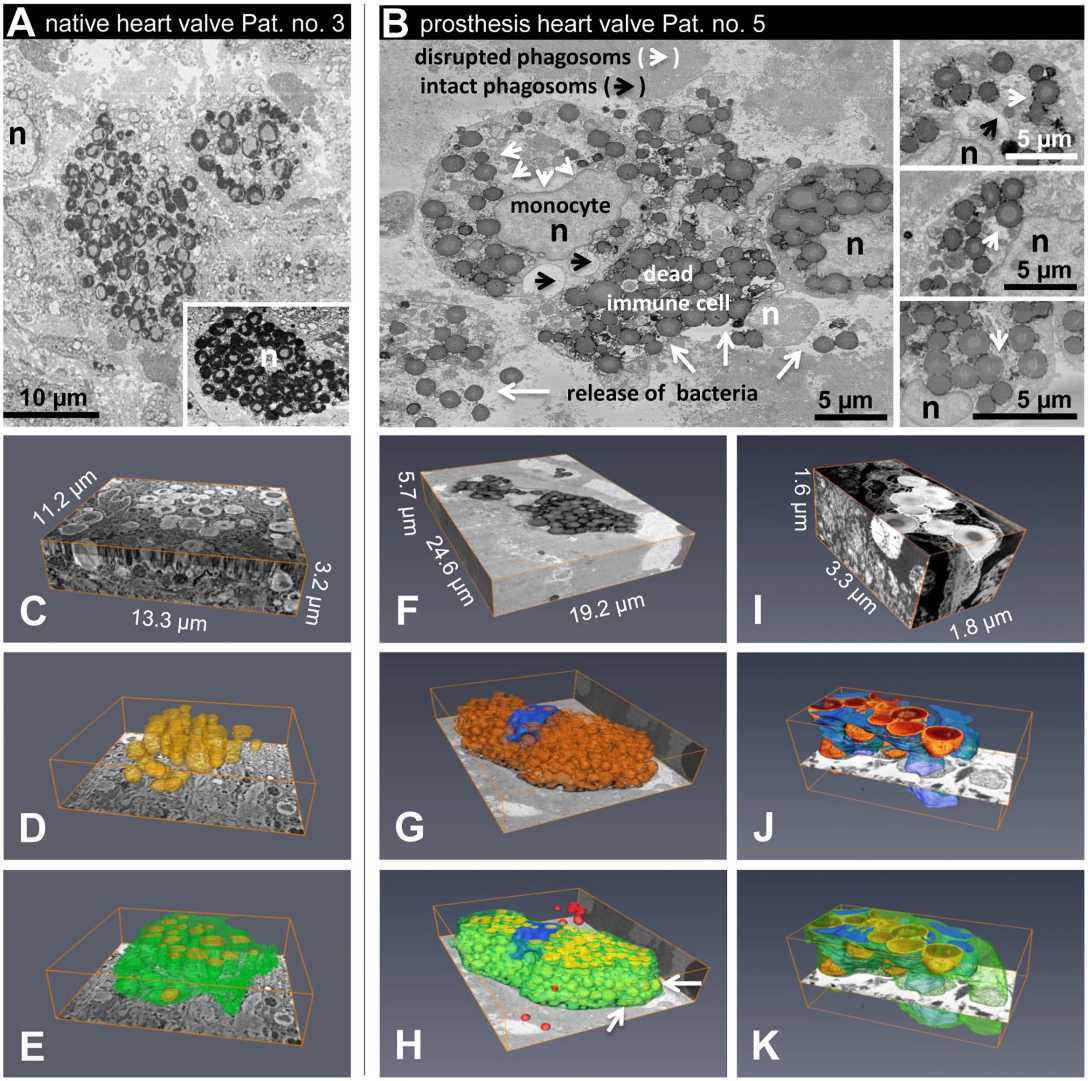
Ultrastructural features of infective endocarditis. Using focused ion beam scanning electron microscopy (FIB-SEM), 3D reconstructions of infected phagocytic cells were generated. (A) Transversal cut through a native infected valve (patient 3). Cells cluttered with numerous viable bacteria showed evidence of a process of intracellular bacterial survival. Nuclei of human cells are indicated by n (black or white). Note some cells carrying a large number of bacteria that nearly fill the cytoplasmic space. Survival of these cells would be unlikely. Inset: a further example from another area. (B) Transverse cut through a biological prosthesis infected with *Staphylococcus aureus* (patient 5). Monocytes cluttered with numerous viable bacteria show evidence of a process in which bacteria escape from a phagocytic vacuole into the cytoplasm; the plasma membrane of some cells is partially disrupted (white arrows), indicating cell death and release of bacterial cargo. Nuclei of monocytes showing intact plasma membrane are indicated by n (black), and the nucleus of a heavily damaged immune cell is indicated by n (white); arrowheads denote phagosomes with intact (black) and disrupted membranes (white); white arrows indicate disrupted plasma membranes. Insets: further examples from another area. (C-E, patient 3) 3View-SEM 3D-reconstruction of a 11.2 μm x 13.3 μm x 3.2 μm (xyz) block at 10 nm x 10 nm x 50 nm (xyz) resolution showing two cells cluttered with numerous viable bacteria (yellow); yellow = bacteria, green = cell membrane. (F-H, patient 5) 3View-SEM 3D-reconstruction of a 24.6 μm x 19.2 μm x 5.7 μm (xyz) block at 10 nm x 10 nm x 50 nm (xyz) resolution showing a monocyte cluttered with numerous viable bacteria (yellow); note that the plasma membrane is partially disrupted (arrows), indicating death of the cell. In addition, several bacteria are located within the extracellular space (red); yellow = bacteria, green = cell membrane, blue = nucleus. (I-K, patient 5) High resolution (2.5 nm x 2.5 nm x 5 nm (xyz)); (F) the nucleus of the cell is highly fragmented as is typical for neutrophilic granulocytes.

Ultrastructural transverse cuts of native and prosthetic specimens featured bacterial clusters and scattered localization pattern (Fig 2A and 2B). At high resolution, the majority of bacteria were localized within phagocytic immune cells, identified as monocytes and neutrophil granulocytes by size and the characteristic structural features of their nuclei (Fig 2B, 2F–2H). In addition to cells cluttered with numerous viable bacteria and apparently intact morphology, many cells were detected with plasma membranes partly disrupted, indicating cell death (Fig 2B and 2H). Although released bacteria seemed to be phagocytized by intact monocytes, ultrastructural analysis showed evidence of a process of bacterial escape from phagocytic vacuoles into the cytoplasm (Fig 2B), which may represent a mechanism to circumvent host immune response and enable survival and persistence inside infected cells. Using the most recent 3View-FIB-SEM 3D-reconstructions, intracellular and intramural localization of bacteria was observed in infected valve tissue (Fig 2C–2K). Intracellular and intramural localization of infective bacteria may complicate successful culture-based microbial standard diagnostics. Therefore, we examined whether the application of culture-independent, DNA-based metagenome analysis might be an appropriate method to overcome such difficulties.

### Individual genetic fingerprints

**Fig 3:**
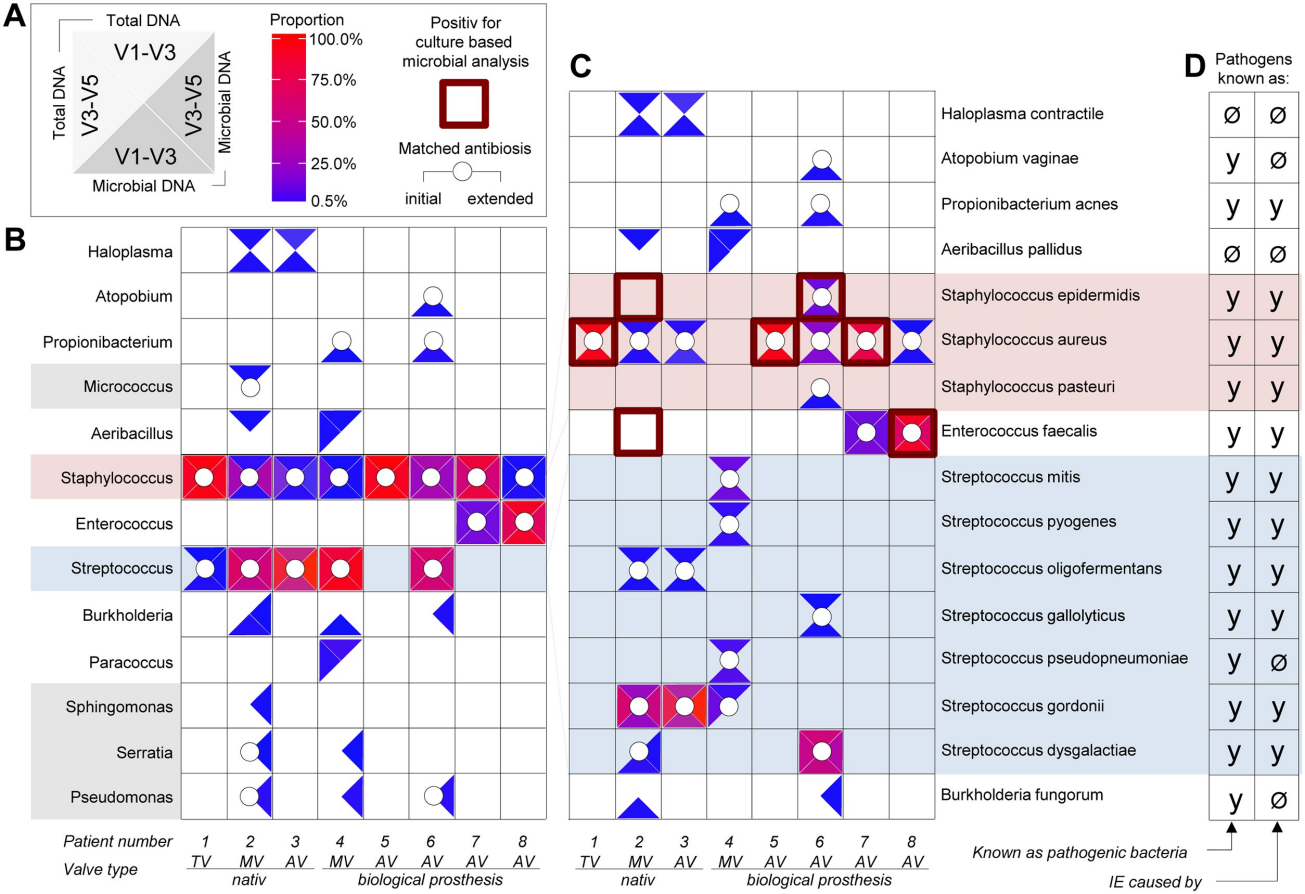
Metagenome analysis of 8 valves obtained from patients with acute infective endocarditis. After surgical valve replacement, total DNA was prepared from resected infected heart valve tissue of IE patients, with microbial DNA being additionally enriched. Subsequently, two hypervariable regions of bacterial 16S rDNA were amplified, Illumina paired-end sequencing libraries were constructed, and next-generation sequencing was carried out using Illumina Miseq technology. The quality of reads obtained was controlled using fastqc and adapters, and low-quality bases were trimmed using Trimmomatic. Remaining reads were aligned to NCBI database 16SMicrobial using software blastn in the megablast mode. Local alignment was repeated with minimum identity filters at 99%. Blast results where then analyzed with MEGAN5. Taxonomic classification was summarized at the species level and further analyzed using statistics software R. Species and genus results were standardized as a proportion of all aligned reads, and cut-off levels of at least 0.5% of species/genus-specific reads were applied. The legend shown in panel A also applies to panels B and C. Bacterial species and genera detected are indicated by colored triangles within a white square. The location of the triangles illustrate the underlying analysis used: **top**—16S rDNA V1-V3 region was amplified and sequenced from total DNA; **left**—16S rDNA V3-V5 region was amplified and sequenced from total DNA; **right**—16S rDNA V3-V5 region was amplified and sequenced from microbial enriched DNA; **bottom**—16S rDNA V1-V3 region was amplified and sequenced microbial enriched DNA. The abundance of genus- or species-specific DNA determined from sequence analysis is indicated in each triangle using a color scale starting from blue (0.5% proportion) to red (100% proportion). Positive culture-dependent identification of bacterial species is highlighted by red squared frames and used in panel C. Matched antibiosis (initial and extended) given prior to surgical intervention is denoted by a white circle. Bacterial genera (B) and species (C) identified in each of the individual patients (numbered) suffering from acute IE are shown. The red-highlighted square frames denote species identified in culture-based microbial analysis. (D) It is shown whether individual bacterial species identified are known and have been previously described as an IE-related pathogen (y = yes) or not (Ø = no). TV = tricuspid valve; MV = mitral valve, AV = aortic valve.

The 16S rDNA NGS analysis successfully revealed bacterial pathogens in all patients of the cohort using total DNA and microbial-enriched DNA (Fig 3A–3C). With this technique, we identified a total of 13 bacterial genera from resected patient valves, including 8 genera representing 16 species. In 5 genera (*Micrococcus*, *Paracoccus*, *Sphingomonas*, *Serratia*, and *Pseudomonas*), identification by DNA analysis did not succeed at the species level. Furthermore, all patient specimens showed bacterial DNA of the *Staphylococcus* genus, and in only one patient (patient 5) could the *Staphylococcus* species not be identified. Aside from 11 well-known IE-related pathogens, our findings revealed several species such as *Haloplasma contractile*, *Atopobium vaginae*, *Aeribacillus pallidus*, *Streptococcus pseudopneumoniae*, and *Burkholderia fungorum* that might play a role as IE-related pathogens (Fig 3D). Two species (*Haloplasma contractile and Aeribacillus pallidus*) have not been previously identified in human infections (S1 Table). The presence that species was verified by specific qPCR analysis (S6 Fig).

In summary, we identified 7 out of 8 cases as being polymicrobial (2–8 species and 2–9 genera per patient), and only one patient showed infection by a single pathogen (*Staphylococcus aureus*) only (Fig 3B and 3C). By additionally performing specific qPCR analysis of selected bacterial species that we initially found to have high or low abundance by 16S rDNA NGS, we validated our sequencing results and further confirmed the hypothesis of polymicrobial infection (S6 Fig). In contrast, standard culture-based microbial detection revealed only single-pathogen infections in 7 out of 8 cases, with one case being negative (Table 2). Of note, bacterial species identified by culture techniques correlated in 5 out of 6 cases with one of those being identified by DNA analysis.

None of the bacterial species successfully cultivated from patient valves or blood in our cohort showed antibiotic resistance (data not shown). The antibiotic treatment protocol, either as initial empiric therapy or extended medication, covered all identified pathogens in 4 out of 8 cases (Fig 3B and 3C); however, the antibiotics chosen for therapy did not cover the entire bacterial spectra identified by our DNA analysis in 4 out of 8 cases and therefore might not have been sufficient (Fig 3B and 3C).

## Discussion

IE remains a major medical issue, with an unchanged incidence and mortality rate over the past 20 years [11, 25]. The mortality rate of 20–30% within the first year of diagnosis and a 5-year mortality rate of 40% are a result of the failure of primary therapy as well as re-infection of prosthetic valves [26, 27]. Despite the myriad of antibiotic regimens currently available (the Federal Institute for Drugs and Medical Devices BfArM states that there were 2,775 approved antibiotics in 2005), apparently adequate treatment does not always result in a cure. It is unclear whether the aforementioned suboptimal outcomes are a result of antibiotic therapy failing to adequately treat the causative organism or whether the causative organism was not accurately identified, thus resulting in inappropriate antibiotic selection.

Interestingly, infections by single bacterial species are described in 99% of all IE cases, based on clinical studies that used standard microbiological approaches [6]. The bacterial species identified were considered to be the causative organism, and therefore antibiotic therapy would be suitably tailored towards it. The origins of pathogens responsible for IE, however, are primarily thought to stem from systemic bacteremia as a result of lesions or surgical interventions of skin or mucosa, leading to barrier leakage and bacterial invasion. One would expect that due to the diversity of bacteria colonizing the skin, gut, and oral mucosa there should be a far higher incidence (than the currently reported 1%) of polymicrobial infections [6]. In the initial phase of infection, blood seems to act as an essential transport vehicle to transfer causative organisms from an unknown infective focus to the heart, causing local valve infection; however, it remains unclear whether the bacterial spectra observed in blood correspond to the spectra of infected valve tissue. If conventional therapy fails and patients demonstrate impaired valve function, surgical intervention with valve replacement is mandatory. This allows accurate tissue diagnosis from the infected valve and further tailoring of antibiotic regimens to prevent prosthetic valve endocarditis by reinfection.

Standard clinical analyses are geared towards the identification of a single pathogen. Major society guidelines recommend blood analysis based on culturing methods to identify the causative organism. In our randomized cohort study of 8 patients with definite acute IE of native valves (3 cases) and biological prostheses (5 cases), we initially analyzed blood profiles. Blood cultures were positive in 7 of our patients and identified as single bacterial infection only (3 species from 2 genera) (Table 2). The success of this method strongly depends on the bacterial ability to grow under artificial culture conditions. Not surprisingly, this is known to fail in 10–48% of patients [6, 12]. The sensitivity is especially limited when antibiotic therapy had been commenced prior to blood drawing and when fastidious or slowly growing microorganisms are involved.

Using standard microbial analysis, only 1 out of 8 valve surface swabs revealed bacterial infestation (*Staphylococcus aureus*) in our cohort. The low sensitivity of only 12.5% is in line with the results of previous studies that reported sensitivities between 8% and 31% [28, 29]. This is surprising because the valve tissue directly represents the infected focus of the organ (in contrast to blood samples). Our FIB-SEM-based ultrastructure analysis of valve surfaces revealed areas exhibiting smooth structures indicative of native valves without evidence of bacterial colonization or areas containing damaged bacteria or debris and only rarely areas displaying potentially intact bacteria (Fig 1). As a consequence, we conclude that valve-surface swabs may largely fail to take up sufficient bacteria retaining the ability to grow. This finding is further supported by the observation that valve pretreatment with ultrasound raised the sensitivity of culture diagnoses to 50% (3 additional positive cases: *Staphylococcus epidermitis*, *Streptococcus gordonii*, *Enterococcus faecalis*). Moreover, ultrastructural analysis of infected biological prostheses revealed significantly bulky surface structures that might be due to the processing and decellularization of animal pericardial tissue used for prosthesis construction. These cavity-like structures might enhance the potential of bacteria to successfully colonize and allow establishment of a niche ecosystem.

To evaluate the possibility of polymicrobial infections in IE, the present study assessed infective bacterial spectra using a modern genetic approach. Techniques such as 16S rDNA analysis allow culture-independent analysis of bacterial infections, but are not yet part of routine laboratory testing. This method is based on total DNA extraction from clinical samples, universal amplification of bacterial 16S rDNA, sequencing of the resulting PCR amplicons, and alignment of sequences using microbial databases to identify bacterial species. Using 16S rDNA NGS, we identified a total of 13 bacterial genera and 16 species from resected patient valves using (Fig 3). In contrast, culture-based valve and blood diagnosis revealed only 2 bacterial genera and 3 species (Table 2). Aside from the 11 well-known IE-related pathogens, 5 species not previously correlated with IE were identified. These included *Haloplasma contractile*, *Atopobium vaginae*, *Aeribacillus pallidus*, *Streptococcus pseudopneumoniae*, and *Burkholderia fungorum*. Information regarding the pathogenicity of these organisms is scarce (S1 Table). Furthermore, mixed infections by *Staphylococci* with either *Streptococci* or *Enterococci* were demonstrated in resected heart valves in 7 of our patients, with a high abundance of specific DNA read counts. *Haloplasma*, *Aeribacillus*, *Paracoccus*, *Propionibacterium*, *Atopobium*, *Micrococcus*, *Burkholderia*, *Serratia*, *Pseudomonas*, and *Sphingomonas* were also identified at lower abundant read levels. In some cases bacterial DNA enrichment was necessary for successful detection. The high sensitivity of 16S rDNA NGS is an advantage that enables the detection of even small traces of bacterial DNA, independent of the organism’s replication performance. In recent years, studies have reported that bacterial pathogenicity depends on endogenous and exogenous factors and are not restricted to bacterial counts alone [30, 31].

With the use of high-resolution NGS, our results support the hypothesis of a complex microbial diversity in IE, with 7 cases being polymicrobial (2–8 species and 2–9 genera per patient) and only one patient with a single pathogen (*Staphylococcus aureus*). The sequencing technique chosen is an important factor in detecting the bacterial diversity of a sample. Almost all previous experimental efforts to analyze infected valve tissue from IE by 16S rDNA used capillary Sanger sequencing, and hence these studies were restricted to the detection of only the single most abundant species [32–35]. Consequently, these studies showed no evidence of infective microbial diversity. Unlike capillary sequencing, however, modern sequencing methods allow the analysis of the entire bacterial community within a sample.

The results of 16S rDNA NGS analysis depend to a varying degree on the use of microbial DNA enrichment procedures, selection of the 16S rDNA region to be amplified, and the number of measurement repetitions. Our estimated recovery rates varied from approximately 80% to 60% in a linear correlation with the cut-off levels of minimum proportion of species-specific sequenced reads from 2% to 0.1%, applied respectively. Our data suggest that the variation in results observed is mainly based on the cut-off levels applied. This means the likelihood of reproducible discovery is much higher for bacterial species present in high abundance (S5 Fig).

Theoretically, in 16S rDNA-based metagenome analysis, PCR amplification of the 16S rDNA target region from multiple bacterial species should take place in parallel under identical conditions within a single assay. But in fact, highly abundant DNA targets compete and therefore suppress the amplification of targets with low abundance. In other words, the spectra of the bacterial species observed tend to be underestimated rather than overestimated. For species with low abundance, the use of a specific qPCR analysis would appear to have a higher sensitivity (S6 Fig). From a clinical standpoint, we propose that species identified with low abundance should be validated by specific qPCR analysis.

Culture-based microbiology was unable to detect pathogens in 4 out of 8 infected valves, whereas, 16S rDNA NGS revealed bacteria in all cases (Table 2 and Fig 3). In comparison with culture-based microbial analysis, the main weakness of the sequencing approaches is the inability to distinguish between living or dead bacteria and the inability to test for antibiotic resistance. Surprisingly, none of the bacterial species successfully cultivated from patient valves or blood in our cohort revealed any relevant antibiotic resistance (data not shown). The antibiotics chosen for therapy, however, did not cover the bacterial spectra identified by our metagenome analysis in 4 out of 8 cases; of these 4 patients, 2 died postoperatively. The clinical condition of the patients made surgical intervention with valve replacement mandatory.

An additional challenge for standard microbial analysis is the detection, identification, and culturing of intracellularly occurring pathogens [36]. In particular, *Staphylococcus aureus*, as identified in 7 out of 8 cases of our cohort, is now recognized as a facultative intracellular pathogen that is able to invade, survive, and replicate intracellularly, e.g. in endothelial and epithelial cells. Using 3D imaging, our data show for the first time proof of intracellular and intramural localization of pathogens in both native and prosthetic heart valves by applying sophisticated FIB-SEM. This microbial strategy may represent an effective mechanism to evade host immune defense and may contribute to the persistence of endocarditis induced by *S*. *aureus* [37]. Even for immune-competent professional phagocytes like neutrophil granulocytes and monocytes, intracellular colonization has been demonstrated [37]. This observation was confirmed by 3D reconstruction using our FIB-SEM data (Fig 2, S1–S3 Movies). In addition to *Staphylococci*, *Streptococci* showed evidence of intracellular cytosolic survival circumventing host immune mechanisms. Intracellular survival of *Streptococci* in polymorphonuclear neutrophils has also been reported in experiments in mice, leading to increased bacterial virulence [38].

The occult localization of certain bacterial species strongly suggests that the standard microbiological diagnosis (e.g. from valve swabs) is not sensitive enough. This creates challenges for a targeted antibiotic therapy. Furthermore, many facultative intracellular bacteria are characterized by impaired growth and reduced metabolism [36]. The effectiveness of antibacterial agents, however, strongly depends on bacterial growth or cellular metabolic activity, with only a few exceptions such as daptomycin and gramicidin [39]. Based on our findings, antibiotic agents must first penetrate the valve surface, then disseminate into tissue, and finally enter both the intact membranes of host cells and bacterial cell walls to be effective. Thus, pharmacokinetic studies should investigate whether antibiotics administered orally or intravenously are properly dosed to result in an effective intracellular cytosolic level.

In conclusion, the high frequency of polymicrobial infections, pathogen diversity, and intracellular persistence of common IE-causing bacteria may provide clues to explain the persistent, devastating mortality rate observed in IE. Improved bacterial diagnosis by 16S rDNA NGS methods resulting in the ability to tailor antibiotic therapy may lead to improved outcomes. Potential caveats, however, include the inability to distinguish between living and dead organisms and the unknown immunogenicity and pathogenicity of species detected in low abundance; these drawbacks remain to be resolved.

## Supporting information

S1 Fig Detection and localization of *Staphylococcus aureus* in infected native and biological prosthetic heart valves. Immunofluorescence staining was performed on cryosectioning tissue of native (A-B) and biological prosthetic (C-F) heart valves using an anti-Staphylococcus aureus specific antibody. Nucleic acids, stained with hexidium iodide (HI), are shown in red; Staphylococcus aureus bacteria are shown in green. White arrows denote bacterial accumulation (Staphylococcus aureus).
(TIF)

S2 Fig Negative control experiments ensure absence of bacterial contamination. Heart valve tissue was homogenized and total DNA was extracted. Subsequently, hypervariable regions of bacterial 16S rRNA gene were amplified as described in material and methods, except 35 cycles (instead of 30 cycles) was used to ensure the absence of specific PCR products in uninfected tissue probes and controls. PCR products are visualized by agarose gel electrophoresis, showing specific bacterial amplicons in infected heart valve tissue only. Samples 1–7 represent infected valve tissue probes from IE patients. Controls 1 to 3 represent non-infected native aorta valve tissue from a human transplant patient (HTX), a bicuspid juvenile patient, and a bicuspid adult patient, respectively. “H2O control” denotes a sample in which an aliquot of water was used in the extraction procedure instead of tissue. “Negative control” denotes that water was used to ensure negative background in PCR analysis.
(TIF)

S3 Fig Key parameters that ensure successful 16S rDNA analysis. (**A**) Comparison of the bacterial 16S rRNA gene sequence (DNA) of different *Staphylococcus* and *Enterococcus* species. 16S rDNA database entries of *Staphylococcus aureus*, *Staphylococcus simiae*, *Enterococcus faecalis*, and *Enterococcus durans* were compared using public alignment software (Lalign). Part of the nucleotide consensus sequence is shown. A hyphen denotes correlating nucleotide consensus positions, whereas a letter denotes diagnostically relevant positions that differ from consensus (representing: A—adenine, G—guanine, C—cytosine and T—thymine). Note that discrimination of bacterial species *Staphylococcus aureus* versus *Staphylococcus simiae* is only possible within the V1-V3 region, whereas *Enterococcus faecalis* and *Enterococcus durans* can only be distinguished in the V3-V5 region. The 16S rRNA gene is visualized as a brown-bordered box; the promoter region is symbolized by a broken arrow; the 9 hypervariable regions are shown as gray boxes numbered V1-V2; black arrows (white bordered) denote primer used for PCR amplification of the V1-V3 and V3-V5 region. (**B**) Total number of sequenced 16S rDNA (reads) per patient amplified from infected resected valve tissue of IE patients. Brown and grey bars denote read numbers that match by alignment with bacterial database entries, whereas the proportion of non-matching reads are shown in black; brown denotes that total DNA (total) was used as a template for amplification, whereas grey represents microbially enriched (enriched) template DNA. V1-V3 and V3-V5 represent the target amplified 16S rDNA target regions. Note that only a small proportion of the amplified DNA did not match for bacterial DNA, suggesting specific target amplification. (**C**) Frequency of single reads identifying different numbers of species matched to 1, 2, 3–20, or 21-all bacterial species. The dependence on different identity parameters (99%, 97% and 95%) is shown for one patient sample. Note that only an accepted identity of 99% precise identification of distinct bacterial species is possible. (**D**) Genetic tree diagram of all bacterial species identified from one patient sample shown as a function of cut-off value reflecting a minimum of 0.1%-10% (shown in different colors) proportion specific reads of the entire population. Note that a cut-off rate of 0.5% gives sufficiently sensitive but robust identification results and reduces background noise.
(TIF)

S4 Fig Standardized workflow used for the identification of valvular bacteria by 16S rDNA by next-generation sequencing. The standardized workflow used for the identification of valvular bacteria by 16S rDNA NGS is shown.
(TIF)

S5 Fig Determination of the recovery rate in 16S rDNA NGS. To determine recovery rate 16S rDNA NGS, repeated analysis was performed with 7 randomly selected DNA samples. (A) Recovery rates as a function of the minimum proportion cut-off levels applied. Recovery rates (%) are shown as colored bars with standard deviation. Bar colors: magenta, dark blue, light blue, green, and yellow represent a minimum proportion of at least 2.0%, 1.0%, 0.5%, 0.2% and 0.1% specific reads of the entire population, respectively. A regression line shows linear correlation with the coefficient of correlation. Numbers of genera and species identified are shown. (B) Genetic tree diagram of all bacteria species identified in each experiment as a function of minimum proportion cut-off levels applied. The colors magenta, dark blue (or red), light blue, green, and yellow of bars and boxes denote minimum proportion of at least 2.0%, 1.0%, 0.5%, 0.2% and 0.1% specific reads of the entire population, respectively.
(TIF)

S6 Fig Detection of selected bacterial species using qPCR analysis and species-specific primers. Heart valve tissue from 7 patients was homogenized and total DNA was extracted. Subsequently, specific regions of genomic bacterial DNA were amplified as described in material and methods using species-specific primers. Serial dilution standards, negative controls, and samples were simultaneously measured in duplicate. As a positive control, DNA from bacterial cultures of *Staphylococcus aureus*, *Enterococcus faecalis*, *Streptococcus gordonii*, *Haloplasma contractile*, *Burkholderia fungorum*, and *Aeribacillus pallidus* was used. (**A**) Comparison between results of 16S rDNA NGS minimal proportion cut-off 0.1% (black/white table) and specific qPCR analysis (colored table) for *Staphylococcus aureus*, *Enterococcus faecalis*, and *Streptococcus gordonii*. Black and white boxes represent positive or negative detection, respectively. Colored boxes denote specific quantitative DNA amounts (ng/ml) as shown in colored standard scale bar. Table numbers indicate patients. Crossed white boxes denote that DNA for qPCR analysis was not available (patient 3). Abbreviations: TV = tricuspid valve; MV = mitral valve, AV = aortic valve. (**B**) Species-specific PCR amplicons (red boxes; fragment length numbered; bp = base pairs) and bacterial DNA targets (grey bars; start and end positions are numbered; bp = base pairs) of *Haloplasma contractile*, *Aeribacillus pallidus* and *Burkholderia fungorum*. The specificity of PCR amplification is shown by plotting the fluorescence as a function of temperature as a melting curve of the amplicon. Grey lines denote melting curves. Reference DNA = pos. control and water = neg. control. Reference accession number numbers and strain IDs are shown. (**C**) Comparison between results of 16S rDNA NGS minimal proportion cut-off 0.1% (black/white table) and specific qPCR analysis (colored table) for *Haloplasma contractile*, *Aeribacillus pallidus*, and *Burkholderia fungorum*. Black and white boxes represent positive or negative detection, respectively. Colored boxes denote specific quantitative DNA amounts (ng/ml) as shown in colored standard scale bar. Table numbers indicate patients. Crossed white boxes denote that DNA for qPCR analysis was not available (patient 3). Abbreviations: TV = tricuspid valve; MV = mitral valve, AV = aortic valve.
(TIF)

S1 Table Characteristics of bacteria previously not reported as being IE associated. (DOCX)

S1 Movie Infected cells in native valve tissue. Using focused ion beam scanning electron microscopy (FIB-SEM), 3D reconstruction of infected cells in native valve tissue (patient 3) was generated. 3View-SEM 3D-reconstruction of a 11.2 μm x 13.3 μm x 3.2 μm (xyz) block at 10 nm x 10 nm x 50 nm (xyz) resolution showing two cells cluttered with numerous viable bacteria (yellow); yellow = bacteria, green = cell membrane.
(MPG)

S2 Movie Infected monocyte in biological prosthesis. Using focused ion beam scanning electron microscopy (FIB-SEM), 3D reconstruction of *Staphylococcus aureus* infected monocyte in biological valve prosthesis tissue (patient 5) was generated. (F-H, patient 5) 3View-SEM 3D-reconstruction of a 24.6 μm x 19.2 μm x 5.7 μm (xyz) block at 10 nm x 10 nm x 50 nm (xyz) resolution showing a monocyte cluttered with numerous viable bacteria (yellow); note that the plasma membrane is partially disrupted (arrows), indicating death of the cell. In addition, several bacteria are located within the extracellular space (red); yellow = bacteria, green = cell membrane, blue = nucleus.
(MPG)

S3 Movie Infected neutrophilic granulocyte in biological prosthesis. Using focused ion beam scanning electron microscopy (FIB-SEM), 3D reconstruction of *Staphylococcus aureus* infected monocyte in biological valve prosthesis tissue (patient 5) was generated. High resolution (2.5 nm x 2.5 nm x 5 nm (xyz)); the nucleus (blue) of the cell is highly fragmented as is typical for neutrophilic granulocytes; yellow = bacteria, green = cell membrane, blue = nucleus.
(MPG)
