# Supplementary figures and images for: New insights into valve-related intramural and intracellular bacterial diversity in infective endocarditis
Source: PLoS One. 2017 Apr 14;12(4):e0175569. doi: 10.1371/journal.pone.0175569 (PMC5391965; doi:10.1371/journal.pone.0175569)

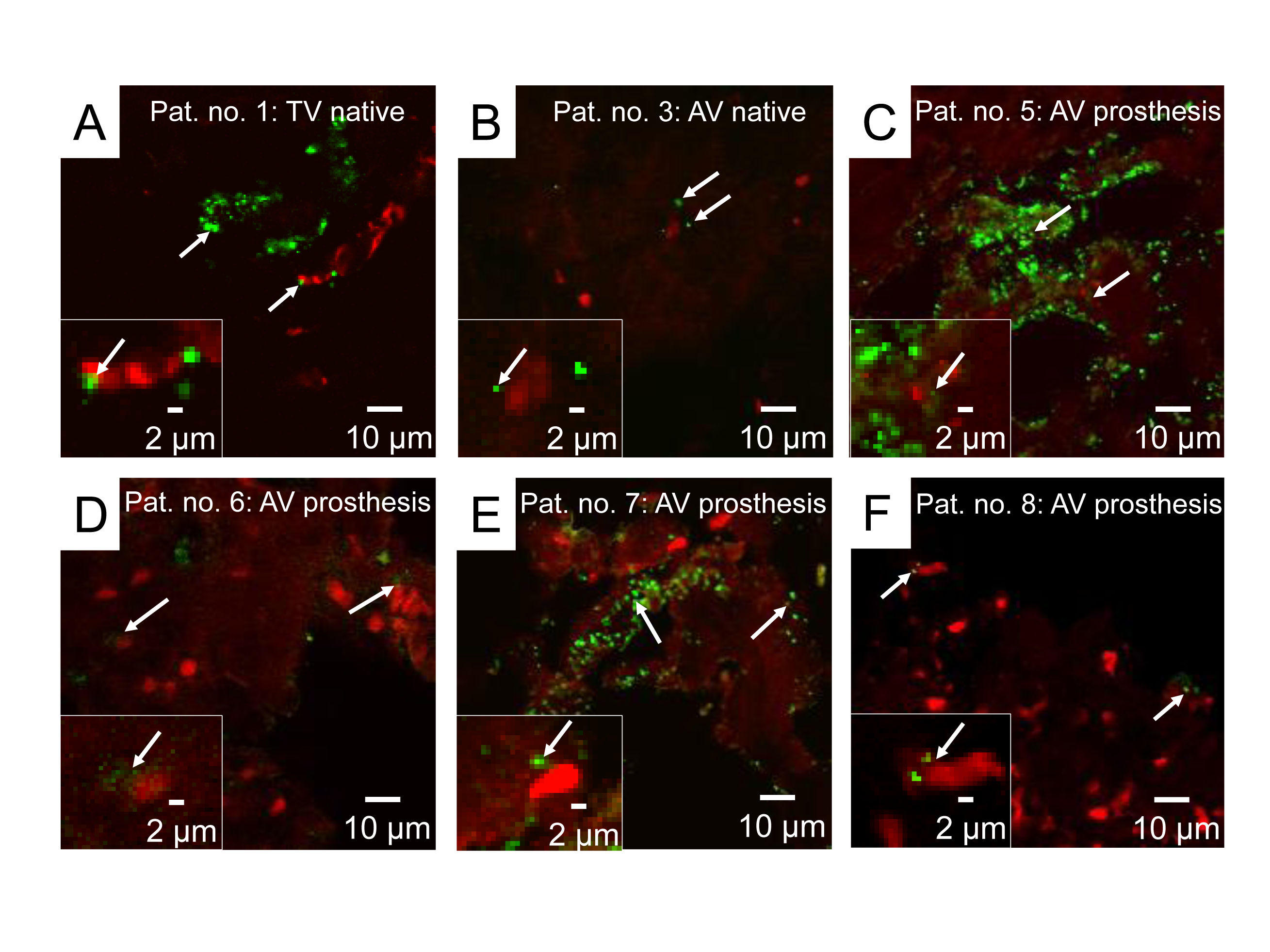

Supplement: S1 Fig — Immunofluorescence staining was performed on cryosectioning tissue of native (A-B) and biological prosthetic (C-F) heart valves using an anti-Staphylococcus aureus specific antibody. Nucleic acids, stained with hexidium iodide (HI), are shown in red; Staphylococcus aureus bacteria are shown in green. White arrows denote bacterial accumulation (Staphylococcus aureus). (TIF) [file pone.0175569.s001.tif]

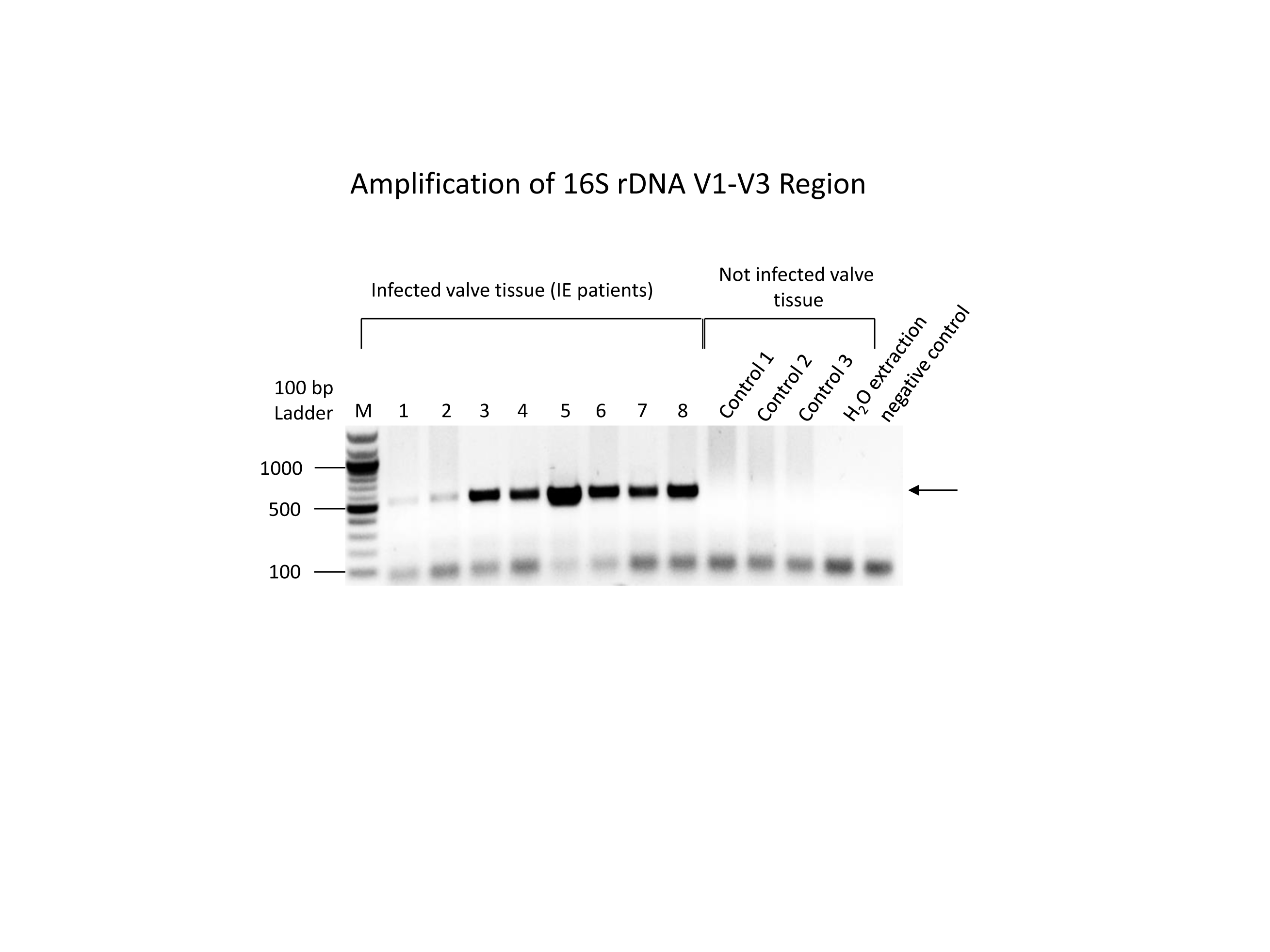

Supplement: S2 Fig — Heart valve tissue was homogenized and total DNA was extracted. Subsequently, hypervariable regions of bacterial 16S rRNA gene were amplified as described in material and methods, except 35 cycles (instead of 30 cycles) was used to ensure the absence of specific PCR products in uninfected tissue probes and controls. PCR products are visualized by agarose gel electrophoresis, showing specific bacterial amplicons in infected heart valve tissue only. Samples 1–7 represent infected valve tissue probes from IE patients. Controls 1 to 3 represent non-infected native aorta valve tissue from a human transplant patient (HTX), a bicuspid juvenile patient, and a bicuspid adult patient, respectively. “H2O control” denotes a sample in which an aliquot of water was used in the extraction procedure instead of tissue. “Negative control” denotes that water was used to ensure negative background in PCR analysis. (TIF) [file pone.0175569.s002.tif]

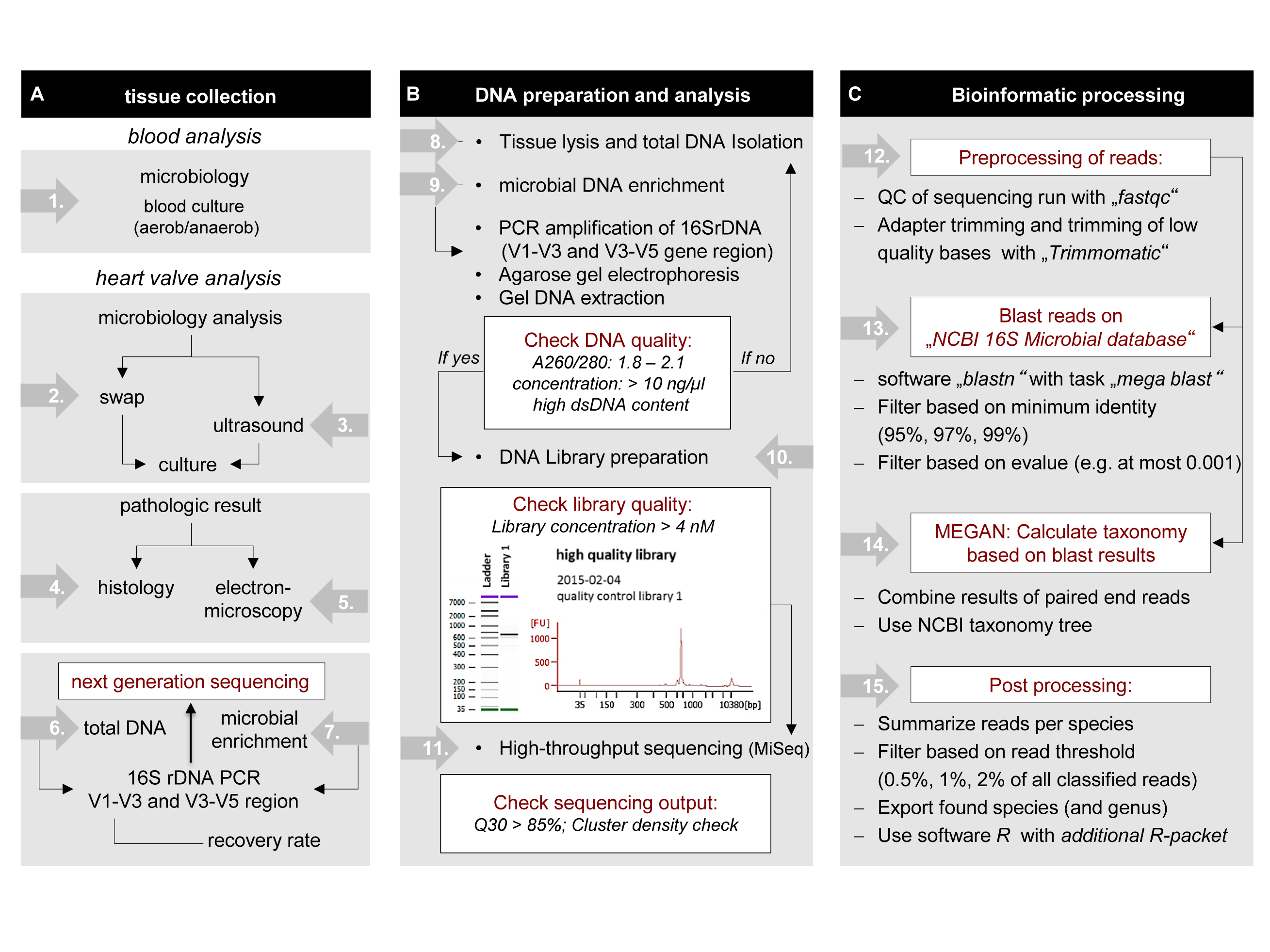

Supplement: S3 Fig — (A) Comparison of the bacterial 16S rRNA gene sequence (DNA) of different Staphylococcus and Enterococcus species. 16S rDNA database entries of Staphylococcus aureus, Staphylococcus simiae, Enterococcus faecalis, and Enterococcus durans were compared using public alignment software (Lalign). Part of the nucleotide consensus sequence is shown. A hyphen denotes correlating nucleotide consensus positions, whereas a letter denotes diagnostically relevant positions that differ from consensus (representing: A—adenine, G—guanine, C—cytosine and T—thymine). Note that discrimination of bacterial species Staphylococcus aureus versus Staphylococcus simiae is only possible within the V1-V3 region, whereas Enterococcus faecalis and Enterococcus durans can only be distinguished in the V3-V5 region. The 16S rRNA gene is visualized as a brown-bordered box; the promoter region is symbolized by a broken arrow; the 9 hypervariable regions are shown as gray boxes numbered V1-V2; black arrows (white bordered) denote primer used for PCR amplification of the V1-V3 and V3-V5 region. (B) Total number of sequenced 16S rDNA (reads) per patient amplified from infected resected valve tissue of IE patients. Brown and grey bars denote read numbers that match by alignment with bacterial database entries, whereas the proportion of non-matching reads are shown in black; brown denotes that total DNA (total) was used as a template for amplification, whereas grey represents microbially enriched (enriched) template DNA. V1-V3 and V3-V5 represent the target amplified 16S rDNA target regions. Note that only a small proportion of the amplified DNA did not match for bacterial DNA, suggesting specific target amplification. (C) Frequency of single reads identifying different numbers of species matched to 1, 2, 3–20, or 21-all bacterial species. The dependence on different identity parameters (99%, 97% and 95%) is shown for one patient sample. Note that only an accepted identity of 99% precise identification o [file pone.0175569.s003.tif]

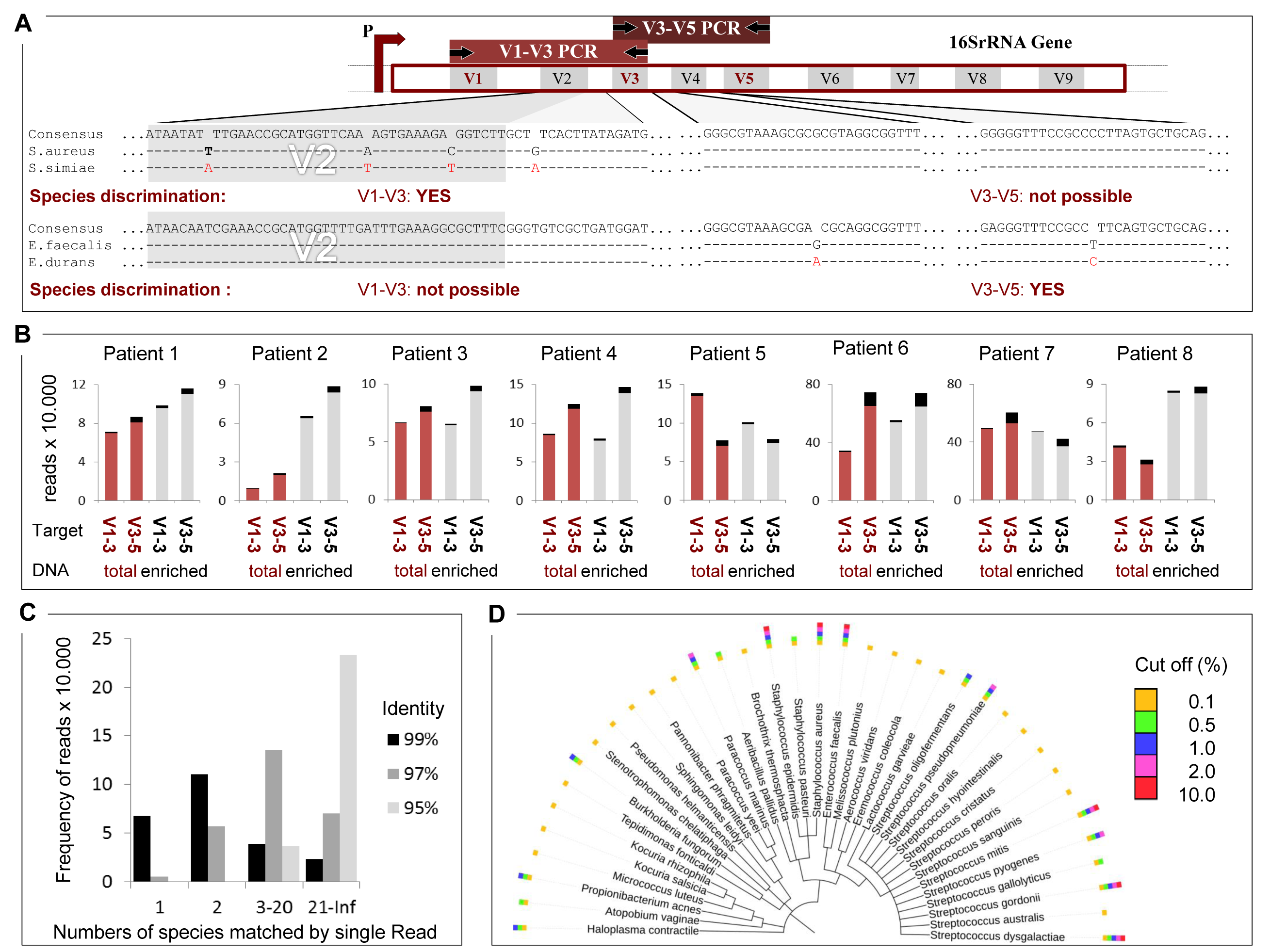

Supplement: S4 Fig — The standardized workflow used for the identification of valvular bacteria by 16S rDNA NGS is shown. (TIF) [file pone.0175569.s004.tif]

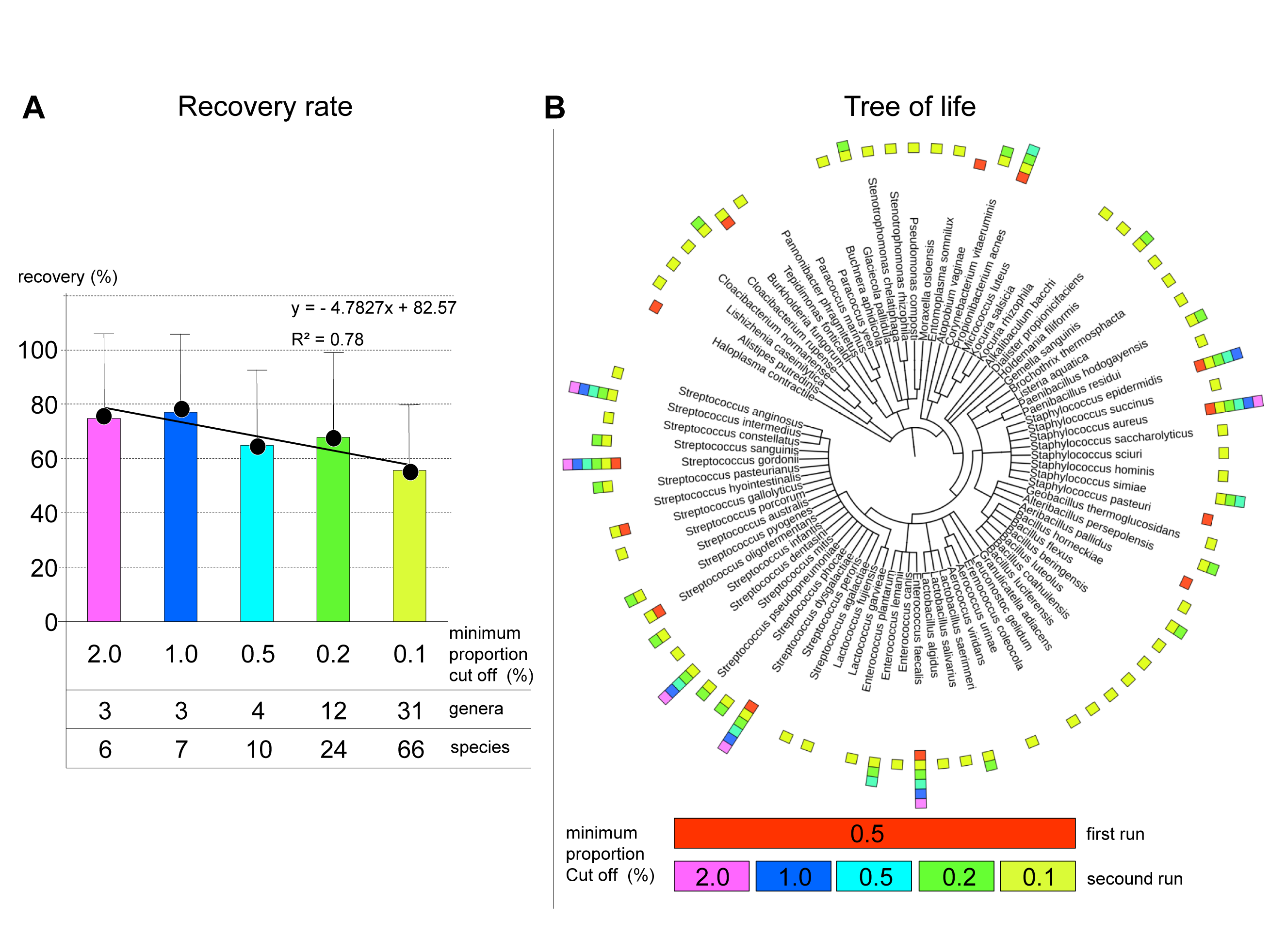

Supplement: S5 Fig — To determine recovery rate 16S rDNA NGS, repeated analysis was performed with 7 randomly selected DNA samples. (A) Recovery rates as a function of the minimum proportion cut-off levels applied. Recovery rates (%) are shown as colored bars with standard deviation. Bar colors: magenta, dark blue, light blue, green, and yellow represent a minimum proportion of at least 2.0%, 1.0%, 0.5%, 0.2% and 0.1% specific reads of the entire population, respectively. A regression line shows linear correlation with the coefficient of correlation. Numbers of genera and species identified are shown. (B) Genetic tree diagram of all bacteria species identified in each experiment as a function of minimum proportion cut-off levels applied. The colors magenta, dark blue (or red), light blue, green, and yellow of bars and boxes denote minimum proportion of at least 2.0%, 1.0%, 0.5%, 0.2% and 0.1% specific reads of the entire population, respectively. (TIF) [file pone.0175569.s005.tif]

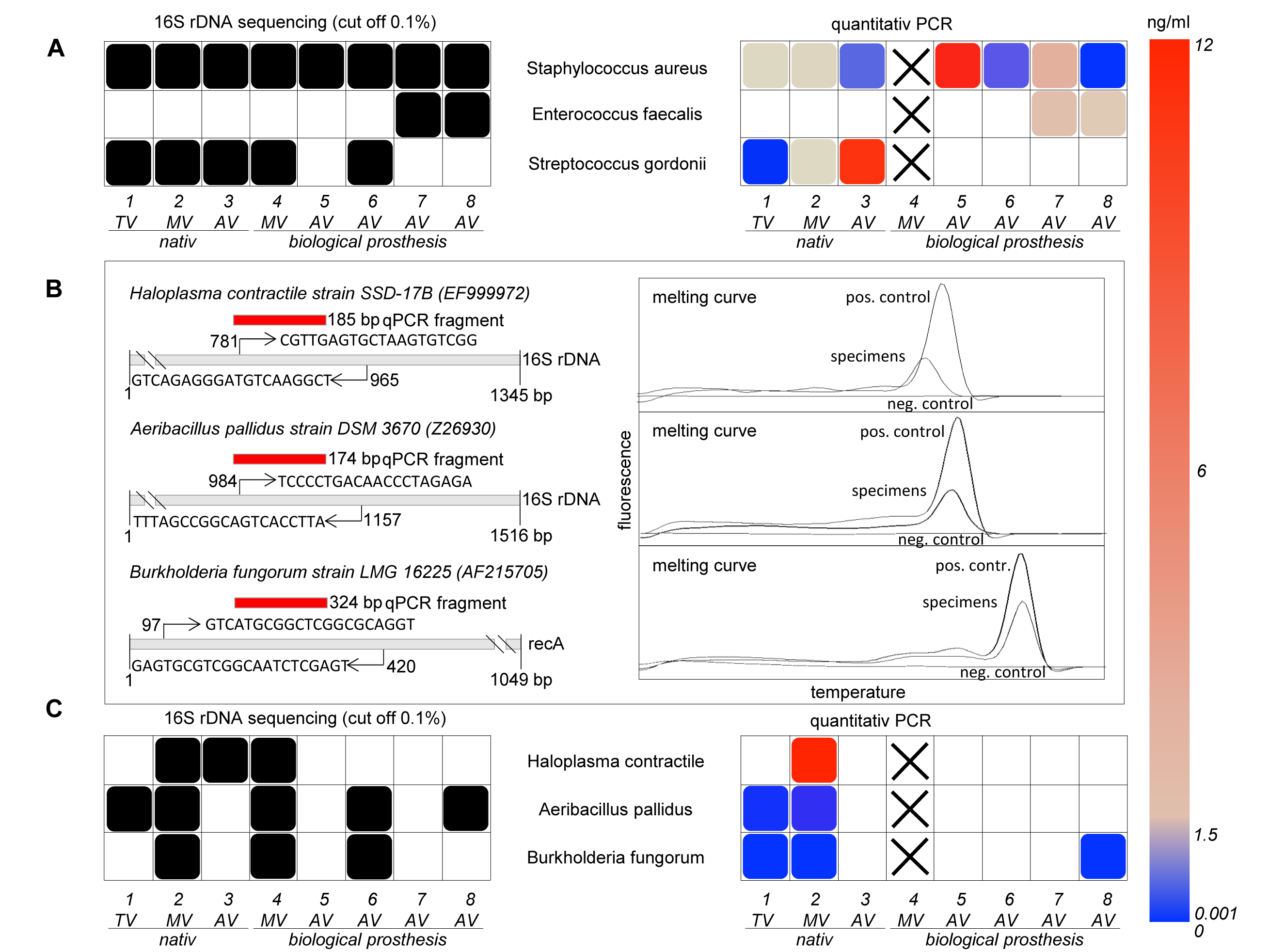

Supplement: S6 Fig — Heart valve tissue from 7 patients was homogenized and total DNA was extracted. Subsequently, specific regions of genomic bacterial DNA were amplified as described in material and methods using species-specific primers. Serial dilution standards, negative controls, and samples were simultaneously measured in duplicate. As a positive control, DNA from bacterial cultures of Staphylococcus aureus, Enterococcus faecalis, Streptococcus gordonii, Haloplasma contractile, Burkholderia fungorum, and Aeribacillus pallidus was used. (A) Comparison between results of 16S rDNA NGS minimal proportion cut-off 0.1% (black/white table) and specific qPCR analysis (colored table) for Staphylococcus aureus, Enterococcus faecalis, and Streptococcus gordonii. Black and white boxes represent positive or negative detection, respectively. Colored boxes denote specific quantitative DNA amounts (ng/ml) as shown in colored standard scale bar. Table numbers indicate patients. Crossed white boxes denote that DNA for qPCR analysis was not available (patient 3). Abbreviations: TV = tricuspid valve; MV = mitral valve, AV = aortic valve. (B) Species-specific PCR amplicons (red boxes; fragment length numbered; bp = base pairs) and bacterial DNA targets (grey bars; start and end positions are numbered; bp = base pairs) of Haloplasma contractile, Aeribacillus pallidus and Burkholderia fungorum. The specificity of PCR amplification is shown by plotting the fluorescence as a function of temperature as a melting curve of the amplicon. Grey lines denote melting curves. Reference DNA = pos. control and water = neg. control. Reference accession number numbers and strain IDs are shown. (C) Comparison between results of 16S rDNA NGS minimal proportion cut-off 0.1% (black/white table) and specific qPCR analysis (colored table) for Haloplasma contractile, Aeribacillus pallidus, and Burkholderia fungorum. Black and white boxes represent positive or negative detection, respectively. Colored boxes denote specific q [file pone.0175569.s006.tif]
