# Supplementary material for: New insights into valve-related intramural and intracellular bacterial diversity in infective endocarditis
Source: PLoS One. 2017 Apr 14;12(4):e0175569. doi: 10.1371/journal.pone.0175569 (PMC5391965; doi:10.1371/journal.pone.0175569)
Supplement: S1 Table — (DOCX) [file pone.0175569.s007.docx]

| **Bacterial species** | **Description** | **Virulence factors** | **Infections in humans** | **Natural habitats** |
| --- | --- | --- | --- | --- |
| **Aeribacillus pallidus** | gram positive rods, aerobic, motile, endospore forming (1) | innovative exopolysaccharides  flagellin protein,  biofilm formation (2,3,4,5) | not reported to date | hydrothermal springs, deep-sea water, oil contaminated soil, bovine milk samples for Aeribacillus spec. (1,4,5,6,7) |
| **Atopobium vaginae** | coccoid gram positive rods, facultaticvely anaerobic, microaerophile, fastidious, non spore-forming, non motile (8,9) | biofilm formation in the vagina (8, 10, 11) | Chronic and recurrent vaginosis, Atopobium spec. described in bacteremia and sepsis in immunocompromised, pelvic inflammatory disease, postpartum endometritis (8,9,11,12,13) | first found in vaginal swab taken from a healthy woman using 16S rRNA analysis (9) |
| **Burkholderia fungorum** | gram negative rods, aerobic, non fermentative, non spore-forming (14) | for B.cepacia complex only: intrinsically resistant to polymyxins, aminoglycosides, β-lactamse exopoly-saccharide, exopolymeric substances (15,16) | septic arthritis, infectious granuloma, intracellular persistence in phagosomes of macrophages (17, 18, 19, 20) | in the environment, in single cases isolated from haemoglobin solution, vaginal secretion of a pregnant woman, CSF of a 66-year-old woman, patient with CF (14,21) |
| **Haloplasma contractile** | Cell-wall-less, pleomorphic-coccoid body with tentacle-like protrusions, anaerobic, fermentative, non-spore-forming (22,23) | unknown | not reported to date | isolated once from deep sea brines (22,23) |
| **Streptococcus pseudopneumoniae** | gram positive cocci (chains, viridans/mitis-group) differentiation from Str. pneumoniae: no bile solubility, optochin resistance, absence of capsule (24, 25) | Pseudopneumolysin, major autolysin, neuraminidase (26) | clinical importance currently unknown, colonization, infection (?) in patients with history of COPD and mainly aspiration pneumonia (24) assumed to be pathogenic | colonizer of respiratory tract, found in sputum samples (24) |

**References:**

1. Yasawong M, Areekit S, Pakpitchareon A, Santiwatanakul S, Chansiri K: Characterization of thermophilic halotolerant Aeribacillus pallidus TD1 from Tao Dam Hot Spring, Thailand.

Int J Mol Sci 2011; 12: 5294–303.

2. Gugliandolo C, Spano A, Lentini V, Arena A, Maugeri TL: Antiviral and immunomodulatory effects of a novel bacterial exopolysaccharide of shallow marine vent origin. J Appl

Microbiol 2014; 116: 1028–34.

3. Naqvi, Syed Aun Muhammad: Production, Structural Characterization And Docking Studies Of Antibacterial Compound From Aeribacillus Pallidus Sat4 Against Prioritized Bacterial

Targets. Pakistan research repository 2013.

4. Radchenkova N, Vassilev S, Panchev I, et al.: Production and properties of two novel exopolysaccharides synthesized by a thermophilic bacterium Aeribacillus pallidus 418. Appl

Biochem Biotechnol 2013; 171: 31–43.

5. Poli A, Anzelmo G, Nicolaus B: Bacterial Exopolysaccharides from Extreme Marine Habitats: Production, Characterization and Biological Activities. Marine Drugs 2010; 8: 1779–802.

6. Oikonomou G, Bicalho ML, Meira E, et al.: Microbiota of cow's milk; distinguishing healthy, sub-clinically and clinically diseased quarters. PLoS One 2014; 9: e85904.

7. Zheng C, Li Z, Su J, Zhang R, Liu C, Zhao M: Characterization and emulsifying property of a novel bioemulsifier by Aeribacillus pallidus YM-1. J Appl Microbiol 2012; 113: 44–51.

8. Backer E de, Verhelst R, Verstraelen H, et al.: Antibiotic susceptibility of Atopobium vaginae. BMC Infectious Diseases 2006; 6: 51.

9. Rodriguez Jovita M, Collins MD, Sjoden B, Falsen E: Characterization of a novel Atopobium isolate from the human vagina: description of Atopobium vaginae sp. nov. Int J Syst

Bacteriol 1999; 49 Pt 4: 1573–6.

10. McMillan A, Dell M, Zellar MP, et al.: Disruption of urogenital biofilms by lactobacilli. Colloids Surf B Biointerfaces 2011; 86: 58–64.

11. Saunders S, Bocking A, Challis J, Reid G: Effect of Lactobacillus challenge on Gardnerella vaginalis biofilms. Colloids Surf B Biointerfaces 2007; 55: 138–42.

12. Oyaert M, Cools P, Breyne J, et al.: Sepsis with an Atopobium-Like Species in a Patient with Fournier's Gangrene. Journal of Clinical Microbiology 2013; 52: 364–6.

13. Cools P, Oyaert M, Vaneechoutte M, Laere E de, Vervaeke S: Atopobium deltae sp. nov., isolated from the blood of a patient with Fournier's gangrene. Int J Syst Evol Microbiol

2014; 64: 3140–5.

14. Coenye T, Laevens S, Willems A, et al.: Burkholderia fungorum sp. nov. and Burkholderia caledonica sp. nov., two new species isolated from the environment, animals and human

clinical samples. Int J Syst Evol Microbiol 2001; 51: 1099–107.

15. Loutet SA, Valvano MA: Extreme Antimicrobial Peptide and Polymyxin B Resistance in the Genus Burkholderia. Frontiers in Cellular and Infection Microbiology 2011; 1: 6.

16. Messiaen A, Forier K, Nelis H, Braeckmans K, Coenye T, Kaufmann GF: Transport of Nanoparticles and Tobramycin-loaded Liposomes in Burkholderia cepacia Complex Biofilms.

PLoS One 2013; 8: e79220.

17. Chiu CH, Ostry A, Speert DP: Invasion of murine respiratory epithelial cells in vivo by Burkholderia cepacia. J Med Microbiol 2001; 50: 594–601.

18. Martin DW, Mohr CD: Invasion and intracellular survival of Burkholderia cepacia. Infect Immun 2000; 68: 24–9.

19. Gerrits GP, Klaassen C, Coenye T, Vandamme P, Meis JF: Burkholderia fungorum septicemia. Emerg Infect Dis 2005; 11: 1115–7.

20. Zhang R, Ran Y, Dai Y, et al.: Infectious granuloma caused by Burkholderia fungorum confirmed by laser-capture microdissection and polymerase chain reaction. Br J Dermatol

2014; 171: 1261–3.

21. Loutet SA, Valvano MA: A decade of Burkholderia cenocepacia virulence determinant research. Infect Immun 2010; 78: 4088–100.

22. Antunes A, Rainey FA, Wanner G, et al.: A New Lineage of Halophilic, Wall-Less, Contractile Bacteria from a Brine-Filled Deep of the Red Sea. J Bacteriol 2008; 190: 3580–7.

23. Antunes A, Alam I, El Dorry H, et al.: Genome sequence of Haloplasma contractile, an unusual contractile bacterium from a deep-sea anoxic brine lake. J Bacteriol 2011; 193:

4551–2.

24. Keith ER, Podmore RG, Anderson TP, Murdoch DR: Characteristics of Streptococcus pseudopneumoniae isolated from purulent sputum samples. Journal of Clinical Microbiology

2006; 44: 923–7.

25. Arbique JC, Poyart C, Trieu-Cuot P, et al.: Accuracy of Phenotypic and Genotypic Testing for Identification of Streptococcus pneumoniae and Description of Streptococcus

pseudopneumoniae sp. nov. Journal of Clinical Microbiology 2004; 42: 4686–96.

26. Johnston C, Hinds J, Smith A, van der Linden, Mark, van Eldere J, Mitchell TJ: Detection of large numbers of pneumococcal virulence genes in streptococci of the mitis group.

Journal of Clinical Microbiology 2010; 48: 2762–9.
